# Validation of left ventricular wall thickening in short-axis cine magnetic resonance imaging by correction of basal-descent through-plane movement

**DOI:** 10.1186/1532-429X-11-S1-P108

**Published:** 2009-01-28

**Authors:** Jyh-Wen Chai, Wei-Hsun Chen, Chih-Ming Chiang, Jachih Fu

**Affiliations:** 1 Taichung Veterans General Hospital, Taichung, Taiwan; 2 Department of Industrial Engineering and Management, National Yunlin University of Science and Technology, Yunlin, Taiwan

**Keywords:** Wall Thickening, Left Ventricular Myocardium, Basal Section, Global Left Ventricular, Left Ventricular Wall Thickening

## Introduction

The problem of the basal descent through-plane movement in quantitative measurement of regional left ventricular (LV) function has been recognized for a long time. But, it is a lack of sophisticated analysis to overcome the limitation.

## Purpose

In this study, we tried to solve the limitation of the through-plane motion in quantification of the regional LV myocardial wall thickness and wall thickening (WT) in the short-axis cine MR imaging.

## Methods

A series of the tagged cine MRI in the long-axis view of left ventricle was acquired to quantize the longitudinal translation of regional myocardium. Based on the quantified longitudinal translation of LV myocardium, a new data set of the LV end-systolic thickness (EST) was reconstructed from the original EST measured in multislice short-axis cine MRI by using a cubic spline interpolating algorithm. The wall thickening (WT) of left ventricle in young volunteers was calculated from the data of the reconstructed EST and the data without correction of the basal descent movement. The results were analyzed and compared to validate the effectiveness of the proposed method in quantification of LV WT.

## Results

The linear correlation between the LVEF and the global LV WT raised from 0.57 before correction to 0.82 after correction of the basal descent through-plane movement. The variability in multislice WT of six segments after correction was significantly smaller than that before correction (0.50 ± 0.05 vs.0.64 ± 0.09, *p* < 0.001). The multislice WT of six segments in the basal sections after correction was significantly different from before correction (*p* < 0.05). The mean regional wall thickening of six radial segments in basal, mid-cavity and apical sections of LV were 42.3 ± 16.8%, 53.3 ± 9.9% and 48.8 ± 30.1% before correction, and 49.4 ± 12.1%, 50.5 ± 12.1% and 53.5 ± 21.3% after correction. The WT in the basal section was much less than that in the mid-cavity section before correction, but was mildly increased and much approximate to that in the mid-cavity section after correction. The results might implicitly imply that the contribution of the myocardium in the basal section to LV function would be as important as that in the mid-cavity.

## Discussion

The proposed technique may be potentially useful in quantification of regional WT of the same level of LV myocardium by correction of the basal descent through-plane movement in SA cine MRI.

